# Prenatal Rosiglitazone Administration to Neonatal Rat Pups Does Not Alter the Adult Metabolic Phenotype

**DOI:** 10.1155/2012/604216

**Published:** 2012-07-03

**Authors:** Hernan Sierra, Reiko Sakurai, W. N. Paul Lee, Nghia C. Truong, John S. Torday, Virender K. Rehan

**Affiliations:** ^1^Department of Pediatrics, Los Angeles Biomedical Research Institute, Harbor-UCLA Medical Center and David Geffen School of Medicine, University of California, Los Angeles, Torrance, CA 90502, USA; ^2^Division of Endocrinology, David Geffen School of Medicine, University of California, Los Angeles, Torrance, CA 90502, USA; ^3^Department of Obstetrics and Gynecology, David Geffen School of Medicine, University of California, Los Angeles, Torrance, CA 90502, USA

## Abstract

Prenatally administered rosiglitazone (RGZ) is effective in enhancing lung maturity; however, its long-term safety remains unknown. This study aimed to determine the effects of prenatally administered RGZ on the metabolic phenotype of adult rats. *Methods*. Pregnant Sprague-Dawley rat dams were administered either placebo or RGZ at embryonic days 18 and 19. Between 12 and 20 weeks of age, the rats underwent glucose and insulin tolerance tests and *de novo* fatty acid synthesis assays. The lungs, liver, skeletal muscle, and fat tissue were processed by Western hybridization for peroxisome proliferator-activated receptor (PPAR)**γ**, adipose differentiation-related protein (ADRP), and surfactant proteins B (SPB) and C (SPC). Plasma was assayed for triglycerides, cholesterol, insulin, glucagon, and troponin-I levels. Lungs were also morphometrically analyzed. *Results*. Insulin and glucose challenges, *de novo* fatty acid synthesis, and all serum assays revealed no differences among all groups. Western hybridization for PPAR**γ**, ADRP, SPB, and SPC in lung, liver, muscle, and fat tissue showed equal levels. Histologic analyses showed a similar number of alveoli and septal thickness in all experimental groups. *Conclusions*. When administered prenatally, RGZ does not affect long-term fetal programming and may be safe for enhancing fetal lung maturation.

## 1. Introduction

Peroxisome proliferator-activated receptor (PPAR)*γ* is a ligand-activated transcription factor that belongs to the superfamily of nuclear hormone receptors [1]. Several studies have evaluated the role of PPAR*γ* in lung maturation, demonstrating its critical significance in stimulating the alveolar epithelial-mesenchymal paracrine signaling pathway [2–5]. Recent studies have also shown that PPAR*γ* agonists such as rosiglitazone (RGZ) significantly enhance lung maturation when administered antenatally. Its efficacy in enhancing pulmonary maturation and neonatal and long-term safety following postnatal administration has also been demonstrated recently [5, 6]. In those studies, lack of any significant impact on the neonatal and long-term metabolic profile of the exposed offspring was demonstrated [5, 6]. However, data on the long-term effects of RGZ are sparse, and to date no study has examined the effects of RGZ on the metabolic profile of *adult* rats when administered prenatally. 

Despite the morbidity and mortality associated with bronchopulmonary dysplasia (BPD), there are no effective pharmacologic preventive or therapeutic options available. Antenatal steroid administration is the standard of care for augmenting pulmonary maturity in the presence of imminent premature labor [7, 8]. However, steroids have both limitations and concerning side effects [9]. Given that antenatal PPAR*γ* administration enhances lung maturation and may be an alternative to antenatal steroids, it is critically important to determine its long-term safety before this treatment modality can be considered for human use. Therefore, we wanted to determine the adult metabolic profile and lung structure of adult rats exposed to RGZ antenatally and compare these to the metabolic profile and lung structure of rats exposed to dexamethasone antenatally. To accomplish this, we utilized a previously described animal model to study the effects of prenatally administered RGZ on markers of lung maturation and the metabolic programming [5, 6]. 

Based on previous studies, we hypothesized that a PPAR*γ* agonist given prenatally to accelerate lung development would not significantly alter the metabolic profile or phenotype [10]. Given the known effects of PPAR*γ* agonists on the regulation of insulin and lipid metabolism, we examined the effects of antenatal RGZ on the basic metabolic profile by measuring body weight, glucose and insulin tolerance tests, *de novo* fatty acid synthesis, plasma troponin-I, cholesterol, triglycerides, insulin, and glucagon levels [11–13]. Lung maturation in adult animals was assessed by examining the expression of surfactant proteins B (SPB) and C (SPC), PPAR*γ* and ADRP, key alveolar epithelial, and mesenchymal molecular markers [5, 14]. Lung morphometry was assessed by determining radial alveolar counts and septal thickness. 

## 2. Methods

Pathogen-free, time-mated, first-time pregnant Sprague-Dawley rats (285–295 g) were obtained at day 16 of gestation (day 21= term). They were allowed food and water *ad libitum* in a humidity- and temperature-controlled room on a 12-h : 12-h-light : dark cycle. Rats were assigned to each of the 4 treatment groups, receiving either diluent, (cottonseed oil), 0.3 mg/kg of RGZ (Cayman Chemicals, Ann Arbor, MI), 3 mg/kg of RGZ, or 0.25 mg/kg of dexamethasone (Dexa) intraperitoneally (i.p.). The diluent, RGZ or Dexa, was administered using a microsyringe in 100 *μ*L volumes injected i.p. once daily on gestational days 18 and 19, 24 hours apart, for a total of two doses each. On day 22 of pregnancy, the dams delivered spontaneously. A total of 33 pups from 4 litters (for each study group), with a minimum of 2 males and 3 females in each group were studied. Pups were breast-fed *ad libitum* and then weaned to rat chow on postnatal day 21. Glucose tolerance and insulin tolerance tests were performed at 12 weeks of age. To perform these studies, either glucose or insulin was administered after an overnight fast. At 20 weeks, the left lungs were collected and flash-frozen for later Western hybridization to determine the expression of PPAR*γ*, ADRP, SPB, and SPC. Right lungs were inflated with saline at a pressure of 20 cm H_2_O, after which the trachea was immediately ligated to maintain inflation. Subsequently, the lungs were stored in 30% dextrose for 2 weeks after which they were embedded in paraffin for further sectioning, H&E staining, and light microscopy. Liver, muscle, and perinephric fat were also collected and flash-frozen to determine the effects of prenatal RGZ on the expression of PPAR*γ* and ADRP, a downstream target of PPAR*γ*. Peripheral blood was collected and stored at −80°C for later determination of cholesterol, triglyceride, glucagon, insulin, cardiac troponin, and fatty acids. In a subset of animals (*n* = 6 for each group; 3M : 3F), at 19 weeks *de novo* fatty acid synthesis and incorporation into tissues were analyzed by deuterium (D_2_O) labeling and mass spectrometry, as previously described [15]. Briefly, animals received deuterated water (99.9%) prepared in normal saline in an amount equal to ~4% of body weight and administered intraperitoneally and then were given free access to drinking water containing 6% D_2_O for 7 days. At the end of the experimental period (~20 weeks of age), the animals were sacrificed using 0.1 mL euthasol ( = 39 mg pentobarbital sodium, Virbac AH, Ft. Worth, TX) per rat. All animal procedures were performed following the guidelines of the National Institutes of Health for the care and use of laboratory animals and approved by the Los Angeles Biomedical Research Institute, Animal Care and Use Committee.

### 2.1. Western Blot Analysis

Western analysis was performed as described previously [5]. The primary antibodies used included SPB, SPC (1 : 500, Santa Cruz Biotechnology Inc., Santa Cruz, CA), PPAR*γ* (1 : 2000, Alexis Biochemicals, San Diego, CA), and ADRP (1 : 500, Santa Cruz Biotechnology Inc., Santa Cruz, CA). The blots were subsequently stripped and reprobed with anti-GAPDH antibody (1 : 10,000, Chemicon, Temecula, CA), and the protein values were normalized to the amount of GAPDH as an internal control.

### 2.2. Glucose Tolerance Test and Insulin Tolerance Test

Either glucose (1 g/kg body wt, intraperitoneal) or insulin (1 unit/kg, subcutaneous) was administered after an overnight fast. Serum glucose levels were assayed at different time points (0, 15, 30, 60, 120, and 180 minutes) using a glucometer (Home Diagnostics, Fort Lauderdale, FL), according to the manufacturer's protocol.

### 2.3. Cholesterol and Triglyceride Assays

Cholesterol and triglyceride levels were determined using the RAICHEM kit (Cliniqa Corporation, San Marcos, CA, with a dynamic range of 0–600 mg/dl, an intra-assay coefficient of variation of 1.7%), and the Cayman kit (Caymen Chemical Company, Ann Arbor, MI, dynamic range of 0–200 mg/dl, and intra-assay coefficient of variation of 1.34%), respectively, following the manufacturer's protocol.

### 2.4. Plasma Insulin and Glucagon

Plasma insulin was measured using an ELISA kit (detection limit of 0.2 ng/mL and 100% specificity) and glucagon was measured via an RIA kit (detection limit of 20 pg/mL and cross-reactivity with oxyntomodulin : <0.1%) purchased from Linco (Linco Research, St. Charles, MO).

### 2.5. Measurement of Plasma Cardiac Troponin-I Levels

Determination of cardiac troponin-I levels was done based on a rat cardiac Troponin-I ELISA kit as per the manufacturer's protocol (Cat. no. 2010-2-HSP, Life Diagnostics, West Chester, PA) (detection limit of 0.156 ng/mL and 100% specificity).

### 2.6. Fatty Acid Analysis

*De novo* fatty acid synthesis was analyzed by deuterium labeling, followed by mass spectrometry, as previously described [5, 15].

### 2.7. Histologic Analysis

Lung morphometry was performed following previously described methods [10].

### 2.8. Statistical Analysis

Analysis of variance and two-tailed Student's *t*-test with Bonferroni correction for multiple comparisons were used to analyze the experimental data. *P*  values < 0.05 were considered to be statistically significant.

## 3. Results

### 3.1. Effect of RGZ on Body Weight

**Figure 1:**
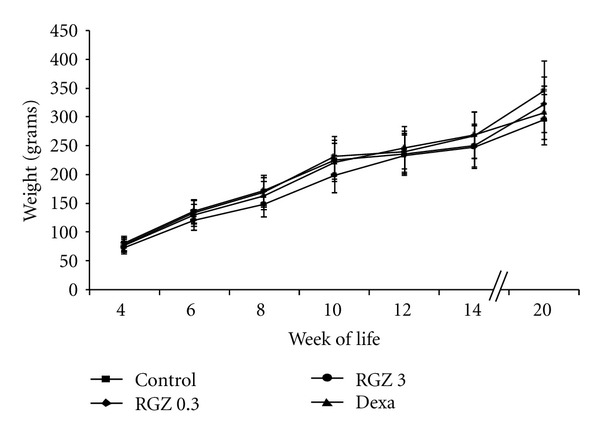
Effect of RGZ on postnatal weight gain. RGZ was administered prenatally at doses of 0.3 mg/kg and 3 mg/kg i.p. ×2 doses. Another group was treated prenatally with i.p. dexamethasone (Dexa, 0.25 mg/kg) ×  2 doses. Weight was recorded every 2 weeks starting at week 4 until week 14 and on the day of sacrifice. There were no significant differences between study groups through out the observation period.

A total of 33 pups (8-9/group) were studied in each group. There were no significant differences in birth weight of pups from each experimental group. Body weight was determined every 2 weeks as an overall measure of growth and metabolism starting on day 30 of life until week 14 and at sacrifice. We found no significant differences (*P* > 0.05) in body weight among the treatment groups at all time-points examined (Figure 1).

### 3.2. Effect of RGZ on Glucose and Insulin Tolerance

**Figure 2:**
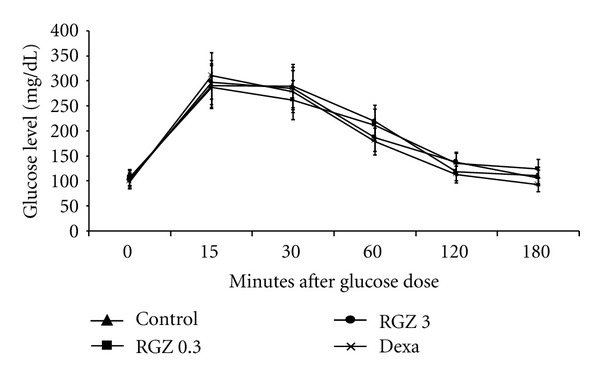
Effect of RGZ on glucose tolerance test (GTT). At 12 weeks of age, glucose was administered at 1 g/kg body weight i.p. after an overnight fast. Glucose was assayed at time = 0 (baseline), 15, 30, 60, 120, and 180 minutes following glucose administration. There were no significant differences (*P* > 0.05) in timed serum glucose values in the treated groups compared with controls during the GTT.

**Figure 3:**
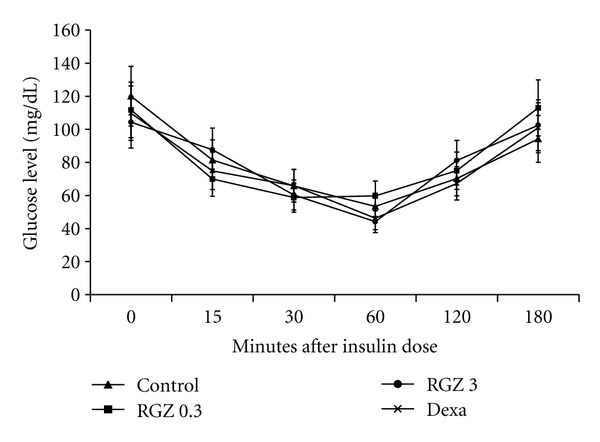
Effect of RGZ on insulin tolerance test (ITT). At 12 weeks of age, insulin was administered at 1 unit/kg body weight subcutaneously after an overnight fast. Glucose was assayed at time = 0 (baseline), 15, 30, 60, 120, and 180 minutes following insulin administration. There were no significant differences in serum glucose values between the treated and control groups during any of the time points of the ITT (*P* > 0.05).

 Glucose and insulin tolerance tests showed no significant differences in glucose values among the different groups at all time-points examined (Figures 2 and 3). 

### 3.3. Effect of RGZ on Insulin, Glucagon, and Cardiac Troponin Levels

Table 1 shows that there were no significant differences in insulin, glucagon, or cardiac troponin-I levels among any of the groups, (*P* > 0.05 for all). 

### 3.4. Effect of RGZ on Blood Cholesterol and Triglyceride Levels

Table 1 shows no significant differences in plasma cholesterol and triglyceride levels in the control group versus the RGZ or Dexa-treated group, (*P* > 0.05 for all). 

### 3.5. Effect of RGZ on Fatty Acid Synthesis. 

Analyses of *de novo* fatty acid synthesis and their incorporation into tissues at 19 weeks showed that the fraction of *de novo* synthesized palmitate molecules in the RGZ- and Dexa-treated groups were comparable to the control group (Table 2).

### 3.6. Effect of RGZ on Alveolar Differentiation

**Figure 4:**
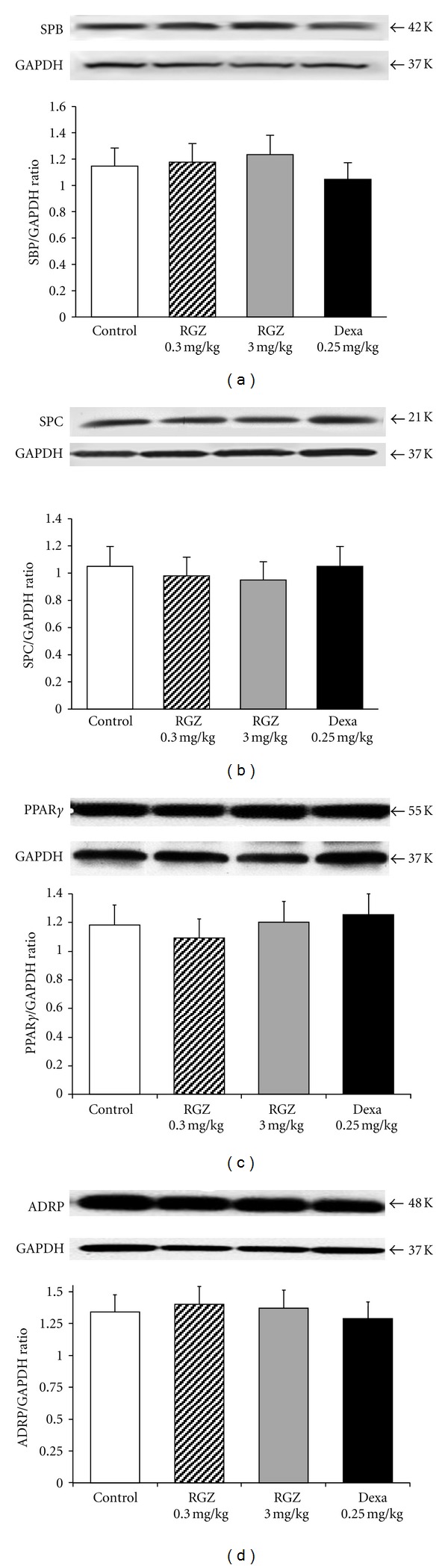
Effect of RGZ on SPB, SPC, PPAR*γ*, and ADRP expression in lung. Utilizing Western blot, SPB, SPC, PPAR*γ*, and ADRP levels were determined in lung lysates. There were no significant differences (*P* > 0.05) in protein levels of SPB (a), SPC (b), PPAR*γ* (c), or ADRP (d) in lung, in the RGZ- and dexamethasone- (Dexa-) treated groups compared with the controls. Representative Western blots and the corresponding density histograms are shown (*n* = 4 in each group).

Western blot analysis for SPB, SPC, PPAR*γ*, and ADRP on protein lysates from whole lung samples from different groups showed that when compared to control, Dexa- and RGZ-treated groups had no significant effect on the expression of all the molecular markers probed (*P* > 0.05 for all, Figure 4).

### 3.7. Effect of RGZ on PPAR*γ* and ADRP Expression in Liver, Muscle, and Perinephric Fat

**Figure 5:**
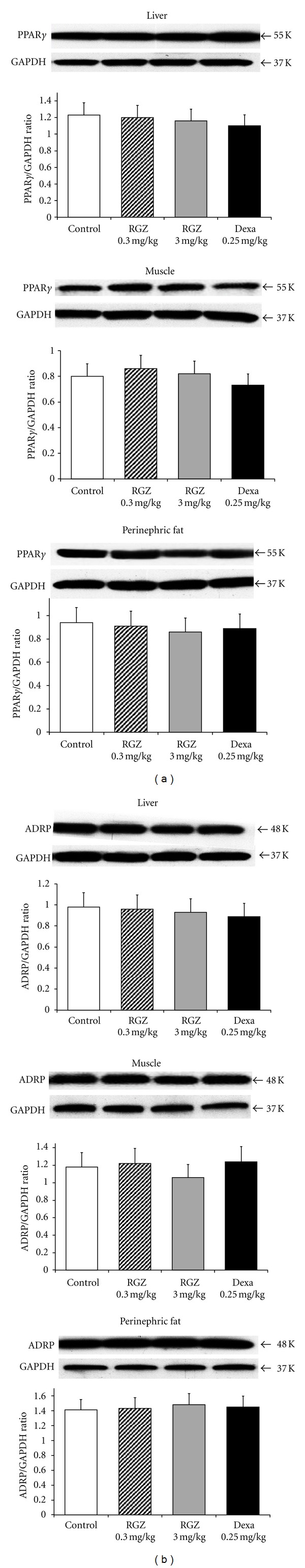
Effect of RGZ on PPAR*γ* and ADRP expression in liver, skeletal muscle, and perinephric fat. Utilizing Western blot assay, PPAR*γ* (a) and ADRP (b) protein levels were examined in the whole tissue lysates of liver, skeletal muscle, and perinephric fat. There were no significant differences (*P* > 0.05) in the protein levels of PPAR*γ* and ADRP in the liver, skeletal muscle, or perinephric fat, normalized to GAPDH, in the treated groups compared with the control. Representative Western blots and the corresponding density histograms are shown (*n* = 4 in each group).

Figure 5 shows Western Blot results for the extrapulmonary PPAR*γ*- and ADRP-expressing tissues (liver, muscle, and perinephric fat) examined. There were no significant differences in PPAR*γ* and ADRP protein levels among the different treatment groups when compared with controls (*P* > 0.05 for all).

### 3.8. Lung Histology

**Figure 6:**
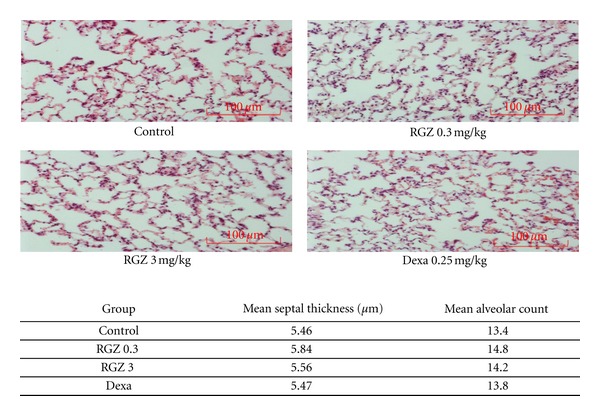
H&E staining of lung samples showed no significant differences in the alveolar number and septal thickness at 20 weeks, between control and treated groups. Light microscopy images of the sections at 20x magnification are shown. *P* > 0.05 for all groups and *n* = 4 in each group.

Morphometric analysis showed no significant differences in septal thickness and alveolar count between the control, Dexa, and RGZ-treated groups (*P* > 0.05, Figure 6).

## 4. Discussion

In view of the increasing survival of extremely low birth weight infants and the accompanying increased prevalence of BPD, it is imperative that we find optimal preventive and therapeutic interventions to decrease the morbidities and mortality associated with this condition [16]. At present, the standard of care to augment lung maturity during imminent premature delivery is antenatal steroid administration; however, evidence suggests steroids may increase the risk for significant adverse effects like altered neuronal development [17]. Despite the necessity to find an optimal treatment for lung immaturity, extensive research in the field has not succeeded in finding such an alternative to antenatal steroids. In the last decade, the possibility of using PPAR*γ* agonists to enhance lung maturation and promote lung injury repair has been explored [1–3]. In addition, our laboratory has shown that in the developing lung PPAR*γ* agonists can prevent lung injury induced by infection, nicotine, or hyperoxia [18, 19]. Similarly, a recent study by Garg et al. has provided evidence that early postnatal administration of PPAR*γ* agonists can reverse the effects of growth restriction [20].

Regardless of its evident efficacy, the long-term safety of prenatally administered PPAR*γ* agonists is unknown. Our present study is the first to examine the long-lasting molecular effects of prenatally administered RGZ, a potent PPAR*γ* agonist. Our results demonstrate that all of the metabolic parameters examined did not change, and RGZ did not alter the adult phenotype of our experimental groups compared with controls. Given RGZ's known effects on insulin and fat metabolism, we determined the body weight patterns across all study groups and observed no significant differences in growth rate and adult weight at 20 weeks of age [12, 19]. Since PPAR*γ* activation regulates the transcription of insulin-responsive genes involved in the metabolism of glucose, we also studied the effects of RGZ on glucose and insulin tolerance as well as glucagon and insulin levels in adults following prenatal RGZ administration [21]. We found that RGZ did not affect either the glucose or insulin tolerance tests, nor the serum insulin or glucagon levels in any of the experimental groups.

Given that PPAR*γ*-related genes are involved in the regulation of lipid metabolism and have effects on the lipid profile, we also determined serum cholesterol and triglyceride levels, as well as *de novo* fatty acid synthesis, among the experimental groups and found no alterations in either serum triglyceride or cholesterol levels when compared to non-treated animals [11]. Results of mass spectrometric analyses did not show alterations in the rate of *de novo* fatty acid synthesis in the experimental groups. 

Rosiglitazone is widely used in the adult population for the treatment of hyperglycemia in diabetes [21, 22]. Recent reports have associated RGZ at a dose of 4 mg twice daily for a period of 20 weeks with an elevated risk of cardiovascular events in this population [23]. We measured cardiac troponin due to its well-established validity as a marker for cardiac injury and to allow for comparison with previous data on rat cardiac function studies [24]. Our study did not reflect any differences in troponin-I levels among the study groups. In contrast to the human data, the absence of cardiotoxicity of RGZ is probably due to much shorter and lower doses used in our animal study compared to much greater exposure in adults (2 doses in our study versus 280 doses in the adult studies).

In addition, we measured the effect of antenatally administered RGZ on the expression of SBP, SPC, PPAR*γ*, and ADRP (the downstream target of PPAR*γ*) in the lung, and in selected extrapulmonary PPAR*γ*-expressing tissues such as the liver, adipose tissue, and muscle. Our results show that when compared to controls, there were no significant differences in the expression of SPB, SPC, PPAR*γ*, or ADRP in either the pulmonary or extra-pulmonary tissues examined. Lastly, morphologic studies did not show any differences in the septal thickness and number of alveoli between the experimental and control groups. 

In summary, long-term followup after prenatal administration of RGZ showed no effects on body weight, insulin and glucagon tolerance tests as well as on insulin, glucagon, triglyceride, cholesterol, or troponin-I levels. In addition, RGZ did not have any effects on fatty acid synthesis or lung morphology, suggesting absence of any long-term metabolic or pulmonary effects following antenatal exposure. 

Among the various thiazolidinediones, RGZ was selected for this study based on extensive clinical experience of others and our studies on its role in perinatal lung maturation [5, 25–27]. The results of this study should be interpreted with caution since given the small sample size, the possibility of a type II error canot be ruled out. However, the promising benefits of thiazolidinediones at the doses used in our studies and the favorable long-term results in the present study strengthen the argument for the use of PPAR*γ* agonists as an effective and safe alternative for the prevention of BPD. 

## 5. Conclusions

RGZ is an effective intervention in the enhancement of lung maturity and the promotion of lung injury repair. Long-term followup of antenatally treated subjects in our study did not show any changes in their metabolic profile or in their phenotype, suggesting that PPAR*γ* agonists are a safe alternative for the prevention and treatment of BPD. Though human studies have shown increased cardiovascular risk associated with RGZ, such adverse effects were not seen in this study, probably due to very different dosing regimens [23, 28]. RGZ is a prototype for the thiazolidinedione group of drugs and our results possibly demonstrate a beneficial class effect suggesting the need for pharmacokinetic and pharmacodynamic studies in humans with the goal of developing this class of drugs as an effective and safe alternative to enhance fetal lung maturation.

## Figures and Tables

**Table 1:** Insulin, glucagon, lipids, and troponin measurements. Plasma samples taken at 20 weeks for metabolic analyses showed there were no significant differences (*P* > 0.05) in insulin, glucagon, lipids, and troponin measurements in RGZ- and dexamethasone-treated groups compared with controls. Values are mean ± SD. *N* = 24 (6 in each group).

Treatment group	Insulin (ng/mL)	Glucagon (pg/mL)	Triglycerides (mg/dL)	Cholesterol (mg/dL)	Troponin (ng/mL)
Control	2.3 ± 1.7	217.3 ± 12	33.2 ± 15	94.4 ± 15	0.18 ± 0.18
RGZ 0.3 mg/kg	2.6 ± 1.4	190.6 ± 6	35.6 ± 6	105.1 ± 15	0.16 ± 0.23
RGZ 3 mg/kg	2.7 ± 0.9	208.2 ± 23	32.5 ± 23	108.9 ± 14	0.10 ± 0.08
Dexamethasone 0.25 mg/kg	2.3 ± 1.4	207.8 ± 13	30.3 ± 13	95.8 ± 21	0.13 ± 0.13

**Table 2:** Effect of RGZ on fatty acid synthesis. At 19 weeks, the fraction of *de novo* lipogenesis and incorporation into the tissues were analyzed by deuterium labeling and mass spectrometry. There were no significant differences (*P* > 0.05) in the fraction of *de novo* synthesis of palmitate molecules in the RGZ- and dexamethasone-treated groups compared with the controls. m1 = fraction of isotopomer molecules with one deuterium substitution, m2 = fraction of isotopomer molecules with two deuterium atoms. *N* = 24 (6 in each group).

Group	m2/m1	Deuterium enrichment (%)	Fraction of new palmitate molecules
Control	0.36	0.04	0.28
RGZ 0.3 mg/kg	0.41	0.04	0.28
RGZ 3 mg/kg	0.35	0.04	0.25
Dexamethasone 0.25 mg/kg	0.37	0.04	0.30
